# SHARPIN is a key regulator of immune and inflammatory responses

**DOI:** 10.1111/j.1582-4934.2012.01574.x

**Published:** 2012-09-26

**Authors:** Zhe Wang, Christopher S Potter, John P Sundberg, Harm Hogenesch

**Affiliations:** a Department of Comparative Pathobiology, Purdue University College of Veterinary MedicineWest Lafayette, IN, USA; b The Jackson LaboratoryBar Harbor, ME, USA

**Keywords:** *Sharpin*, immune system, inflammation, chronic dermatitis, NF-κB, ubiquitination, lymphoid organogenesis, eosinophilic dermatitis, scaly skin disease

## Abstract

Mice with spontaneous mutations in the *Sharpin* gene develop chronic proliferative dermatitis that is characterized by eosinophilic inflammation of the skin and other organs with increased expression of type 2 cytokines and dysregulated development of lymphoid tissues. The mutant mice share phenotypic features with human hypereosinophilic syndromes. The biological function of SHARPIN and how its absence leads to such a complex inflammatory phenotype in mice are poorly understood. However, recent studies identified SHARPIN as a novel modulator of immune and inflammatory responses. The emerging mechanistic model suggests that SHARPIN functions as an important adaptor component of the linear ubiquitin chain assembly complex that modulates activation of NF-κB signalling pathway, thereby regulating cell survival and apoptosis, cytokine production and development of lymphoid tissues. In this review, we will summarize the current understanding of the ubiquitin-dependent regulatory mechanisms involved in NF-κB signalling, and incorporate the recently obtained molecular insights of SHARPIN into this pathway. Recent studies identified SHARPIN as an inhibitor of β1-integrin activation and signalling, and this may be another mechanism by which SHARPIN regulates inflammation. Furthermore, the disrupted lymphoid organogenesis in SHARPIN-deficient mice suggests that SHARPIN-mediated NF-κB regulation is important for *de novo* development of lymphoid tissues.

- Introduction
- *Sharpin* mutations in mice
- SHARPIN in immune development and function
  – NF-κB signalling and regulation through ubiquitination
  – SHARPIN and NF-κB regulation
  – SHARPIN and β1-integrin signalling
  – SHARPIN in lymphoid organogenesis
- Concluding remarks

## Introduction

*Sharpin* (also known as *cpdm*, *sipl1*; see Table 1 for a list of acronyms of factors in signalling pathways used in this review) is an autosomal gene that is conserved among many mammalian species, including humans, mice, rats, dogs, cattle, chimpanzees and non-human primates (NCBI Entrez Gene Database, http://www.ncbi.nlm.nih.gov/gene). *Sharpin* is ubiquitously expressed in various types of cells and tissues. The protein product SHARPIN is localized in the cytoplasm of cells [1], but recent data suggest that it is also present in membrane ruffles and in the nucleus [2]. Domain analysis programs Scansite and InterProScan (http://ca.expasy.org/) predict the existence of multiple functional motifs based on the amino acid sequence of mouse SHARPIN. The amino-terminal coiled-coil domain (CC) is a structural motif (composed of two-five alpha-helices) that is primarily involved in protein-protein interaction [3]. Indeed, the CC domain mediates the interaction between SHARPIN and the scaffold protein SHANK in excitatory synapse of rat brain [1]. The other two domains, ubiquitin-like domain (ULD) and NPL4 zinc finger domain (NZF), facilitate ubiquitin-mediated protein recognition and degradation [4]. The NZF domain supports interaction between the adaptor proteins TAB 2/3 and the poly-ubiquitin chain on TAK1, thereby activating the downstream IκBα kinase and NF-κB signalling cascade [5]. Recent studies have shown that both ULD and NZF domains are essential for SHARPIN to exert its function in part through ubiquitin-mediated mechanisms [6–8].

**Table 1:** Acronyms of factors involved in signalling pathways and their synonyms

Acronym	Full name	Synonyms
A20	Tumour necrosis factor, alpha-induced protein 3	TNFAIP3, TNFIP3
BAFF	Tumour necrosis factor (ligand) superfamily, member 13b	TNFSF13B, Blys
BCL3	B cell leukaemia/lymphoma 3	
cIAP1	Baculoviral IAP repeat-containing 2	BIRC2
cIAP2	Baculoviral IAP repeat-containing 3	BIRC3
c-REL	Reticuloendotheliosis oncogene	REL
HOIL1	RanBP-type and C3HC4-type zinc finger containing 1	RBCK1, HOIL1L
HOIP	Ring finger protein 31	RNF31
IκBα	Nuclear factor of kappa light polypeptide gene enhancer in B cells inhibitor, alpha	NFKBIA
IκBβ	Nuclear factor of kappa light polypeptide gene enhancer in B cells inhibitor, beta	NFKBIB
IκBε	Nuclear factor of kappa light polypeptide gene enhancer in B cells inhibitor, epsilon	NFKBIE
IKKα	Conserved helix-loop-helix ubiquitous kinase	CHUK
IKKβ	Inhibitor of kappaB kinase beta	IKBKB
IRP2	Iron responsive element binding protein 2	IREB2
NEMO	Inhibitor of kappaB kinase gamma	IKBKG, IKKγ
NF-κB	Nuclear factor kappa B	
NIK	Mitogen-activated protein kinase kinase kinase 14	MAP3K14
NFKB2	Nuclear factor of kappa light polypeptide gene enhancer in B cells 2, p100/p49	p100/p52
NFKB1	Nuclear factor of kappa light polypeptide gene enhancer in B cells 1, p105	p105/p50
RELA	v-rel reticuloendotheliosis viral oncogene homolog A	p65
RIP1	Receptor (TNFRSF)-interacting serine-threonine kinase 1	RIPK1
SCF-βTRCP	Beta-transducin repeat-containing protein	BTRC
SHANK1	SH3/ankyrin domain gene 1	
SHARPIN	SHANK-associated RH domain-interacting protein	cpdm, SIPL1
SHEP	Avian reticuloendotheliosis viral (v-rel) oncogene-related B	RELB
TAB 2	TGF-beta activated kinase 1/MAP3K7-binding protein 2	MAP3K7IP2
TAB 3	TGF-beta activated kinase 1/MAP3K7-binding protein 3	MAP3K7IP3
TAK1	Mitogen-activated protein kinase kinase kinase 7	MAP3K7
TNF	Tumour necrosis factor	TNFα
TNFR1	Tumour necrosis factor receptor superfamily, member 1a	TNFRSF1A
TRADD	TNFRSF1A-associated *via* death domain	
TRAF6	TNF receptor-associated factor 6	
TWEAK	Tumour necrosis factor (ligand) superfamily, member 12	TNFSF12
UBC13/UEV1A	Ubiquitin-conjugating enzyme E2N	UBE2N
UBC4/5	Ubiquitin-conjugating enzyme E2D 2	UBE2D2
PTEN	Phosphatase and tensin homology	

### *Sharpin* mutations in mice

Spontaneous autosomal recessive mutations in *Sharpin* were identified in two different mouse strains in the Netherlands, C57BL/KaLawRij (C57BL/KaLawRij-*Sharpin*^*cpdm*^/RijSunJ, hereafter referred to as *Sharpin*^*cpdm*^ mice) and OcB3/Dem (CBy.OcB3/Dem-*Sharpin*^*cpdm-Dem*^) [9]. Both allelic mutations cause a similar phenotype [10]. The mutations introduce an early stop codon in the first exon resulting in premature termination of translation and no functional protein product [10]. At about 4 weeks of age, *Sharpin*^*cpdm*^ mice start to develop extensive inflammation in multiple organs, including the skin, oesophagus, lung and liver [9, 11, 12]. Mouse organs are not all equally affected, suggesting that SHARPIN may have tissue-specific functions. The skin lesions are clinically the most obvious, characterized by epidermal hyperplasia, diffuse ortho- and focal parakeratotic hyperkeratosis, scattered keratinocyte apoptosis and marked dermatitis due to the infiltration of the dermis with eosinophils, neutrophils, mast cells and macrophages with concurrent progressive neovascularization [9]. Secondary lymphoid organogenesis in *Sharpin*^*cpdm*^ mice is severely impaired as indicated by poorly defined separation of B and T cell areas in the white pulp and lack of marginal zones in the spleen, poorly developed lymphoid follicles in lymph nodes (LNs) and nasal-associated lymphoid tissue and absence of Peyer's patches in the intestine of adult mice [13]. *Sharpin*^*cpdm*^ mice have reduced serum IgG, IgA and IgE and nearly undetectable faecal IgA, whereas the concentration of IgM was normal in serum and increased in faecal samples indicating defective isotype switching. This may result from the disrupted architecture of secondary lymphoid tissues [13] or defective CD40 signalling required for B cell activation [6, 14].

The concentration of type 2 cytokines including IL5 and IL13 is increased in the skin and in the supernatants of stimulated splenocytes of the mutant mice, indicating a type 2-skewed inflammatory phenotype [15]. In line with this, interferon gamma (IFNG) secretion from stimulated splenocytes and IFNG-mediated delayed type hypersensitivity are both significantly impaired. Notably, systemic administration of recombinant mouse IL12 to *Sharpin*^*cpdm*^ mice almost completely resolved the dermatitis [15], suggesting that reduction in the physiological production of IL12 contributes to the pathogenesis of the *cpdm* phenotype. Indeed, dendritic cells from *Sharpin*^*cpdm*^ mice are defective in IL12 production in response to TLR ligands, and have impaired T_H_1-polarization capacity when incubated with CD4^+^ T cells [16]. Consistent with the type 2 inflammation, the expression of the chitinase-like proteins CHI3L3 and CHI3L4, carbohydrate-binding proteins that are typically present in type 2 inflammation [17], was significantly increased in the skin of *Sharpin*^*cpdm*^ mice [18]. These proteins are induced by IL4 and IL13 in macrophages and mast cells, and are markers of alternatively activated macrophages [17, 18].

*Sharpin*^*cpdm*^ mice are a potential model to study the pathogenesis of chronic type 2 inflammatory diseases with accumulation of eosinophils, mast cells and alternatively activated macrophages. Eosinophils are believed to play a contributing role in the initiation and progression of asthma [19, 20], atopic dermatitis [21, 22], collagenous colitis [23] and eosinophilic oesophagitis [24], although the underlying mechanisms leading to the pathogenesis remain to be established. Neutralization of IL5 with antibodies or genetic deletion of IL5 significantly reduced the number of cutaneous and circulating eosinophils in *Sharpin*^*cpdm*^ mice, but did not alter the inflammatory phenotype suggesting that eosinophilia may be a secondary response rather than the initiating mechanism [25]. The role of mast cells and macrophages in the development and maintenance of the *Sharpin*^*cpdm*^ dermatitis remains to be investigated.

SHARPIN was initially identified as a scaffold protein in the postsynaptic density within the central nervous system [1]. The protein interacts with the ankyrin repeat domain of SHANK1 [1], a well-defined structural building block protein at excitatory synaptic sites [26]. In spite of this role of SHARPIN in the architecture of the nervous system, the behaviour of *Sharpin*^*cpdm*^ mutant mice appears to be normal. The absence of a neurological phenotype may be due to the shortened life span of *Sharpin*^*cpdm*^ mice, as the dermatitis becomes quite severe by 10–12 weeks of age necessitating killing [9]. This may prevent the development of an overt neurological phenotype. In addition, severe pruritis and frequent scratching associated with the progressive skin disease may obscure other behavioural changes. Recent studies have concentrated on the mechanism by which the *Sharpin* mutation results in chronic inflammation and suggest that SHARPIN is an important regulator of the activation of NF-κB signalling pathways [6, 8, 14, 16, 27, 28].

### NF-κB signalling and regulation through ubiquitination

Mammalian NF-κB is a family of inducible transcription factors that play a central role in diverse aspects of cellular homeostasis, including cell proliferation, survival and apoptosis, as well as lymphoid tissue development and mobilization of innate and acquired immune response [29–31]. Activated NF-κB protein complexes are homodimers or heterodimers comprised of various combinations of the five family members, RELA, RELB, c-REL, NFKB1 and NFKB2, all of which contain a conserved RHD (*N*-terminal REL-homology domain) that facilitates dimerization, nuclear trafficking, DNA binding and interaction with the inhibitory IκB protein. RELA, RELB and c-REL also possess a C-terminal TAD (transcriptional activation domain) that enables downstream gene activation and expression. The IκB proteins, including IκBα, IκBβ, IκBε and BCL3, together with NFKB1 and NFKB2, are signalling inhibitors that restrain NF-κB dimers from nuclear localization and therefore block transcriptional activity.

The NF-κB signalling is generally classified into a canonical and non-canonical pathway depending on the identity of signalling inhibitors [30, 32]. The canonical pathway, primarily triggered by TNF, LPS (lipopolysaccharide) and T and B cell receptors, occurs in most cells as the principal NF-κB pathway. Upon stimulation, the downstream kinase IKK complex composed of two catalytic subunits IKKα, IKKβ and one regulatory subunit, NEMO, is activated, allowing phosphorylation of the IκBα inhibitory protein. Phosphorylated IκBα is then processed by the ubiquitin-conjugating system that adds ubiquitin chains to IκBα to promote its subsequent proteasome-mediated degradation. Ubiquitin chains are covalently attached to one or multiple lysine residues on target proteins, which serve as signals for proteolysis or activation. Release of the IκBα inhibitor liberates the NF-κB heterodimer, mainly RELA/NFKB1 and allows its nuclear translocation for gene transcription. The canonical pathway preferentially enables expression of the target genes involved in production of cytokines and activation of immune cells, which cooperatively govern the immune and inflammatory response [31]. In contrast, the non-canonical pathway is induced primarily by members of the TNF receptor superfamily, including those for B cell activating factor BAFF, lymphotoxin (LT) and CD40 ligand, although they also induce canonical NF-κB activation. The alternative pathway causes phosphorylation and activation of a kinase cascade, NIK and IKK, which in turn phosphorylate the two serine residues of full length NFKB2. Upon phosphorylation, NFKB2 is subject to the cellular ubiquitin ligase machinery. Unlike ubiquitinated IκBα destined for degradation, polyubiquitination of NFKB2 results in partial degradation of the C terminus to yield a p52 fragment with an exposed *N*-terminal RHD domain. The mature p52 interacts with RELB to form a functional transcription factor that moves into the nucleus to activate target gene expression. The non-canonical NF-κB signalling plays an essential role in B cell maturation and activation, as well as in the development and maintenance of secondary lymphoid tissues [31, 33].

The NF-κB signalling is of great importance in mounting rapid and targeted immunity to microbial invaders; however, over-activation causes excessive inflammatory stresses and damage in tissues. Given the double-edged nature of NF-κB signalling in immune system, one can expect that NF-κB is strictly regulated. Various post-translational modifications such as phosphorylation, ubiquitination, acetylation and sumoylation, modulate their activity and specificity by attaching small functional moieties to various signalling components [34]. Among these regulatory mechanisms of NF-κB, ubiquitination is of particular interest because of the emerging appreciation for its important and versatile role in signalling pathways [35–38]. Ubiquitin conjugates occur in various forms that target protein substrate for different destinies. Ubiquitin chains are traditionally thought to form a functional moiety by polyubiquitination through lysine at position 48 of ubiquitin (Lys48), which mark substrates for degradation by the proteosome machinery. As discussed earlier, phosphorylated IκBα in response to LPS stimulation is conjugated with Lys48 polyubiquitination, a process performed by UBC4/5, an E2 ubiquitin-conjugating enzyme that forms a E2-ubiquitin thioester and SCF-bTRCP, an E3 ubiquitin ligase that attaches ubiquitin to a target substrate [39, 40]. This results in the proteolysis of IκBα and activation of NF-κB transcription factors. Ubiquitin chains also contribute to NF-κB regulation by degradation-independent mechanisms, which include Lys63 polyubiquitination that activates protein kinases. For example, LPS stimulation induces the Lys63 polyubiquitination of TAK1, an essential component of the kinase cascade that in turn phosphorylates other downstream targets to trigger NF-κB signalling. This is carried out by the E2 enzyme UBC13 and the E3 ligase TRAF6 [41].

The distinct functions of Lys48- and Lys63-linked ubiquitin chains in dictating signalling cascades result from different molecular configurations [42]. Polyubiquitination through Lys48 and Lys63 does not appear to create conflicting regulatory strategies, but rather their coordinated combination allows fine tuning of NF-κB activation. For instance, TNF-induced trimerization of TNF receptors recruits TRAF2/5 and the ubiquitin ligase cIAP1/2 and TRAF2/5 to the TNFR1 signalling complex and cIAP1/2 attaches Lys63 polyubiquitin chains to the protein RIP1 [43–46]. It remains controversial whether TRAF2/5 also functions as an E3 ligase to target RIP1 [47, 48]. The Lys63 linked chains on RIP1 serve as a platform for IKK activation, although they are not essential [49–51]. The activating polyubiquitin chain, however, can be removed by the ubiquitin-editing enzyme A20, which utilizes its *N*-terminal de-ubiquitination domain to remove the Lys63 polyubiquitin chain and its C-terminal ubiquitin ligase domain to conjugate new Lys48 polyubiquitin chains to RIP1 [52]. The altered pattern of the polyubiquitin chain not only inactivates the kinase RIP1 but also promotes proteosomal degradation effectively abolishing TNF-induced NF-κB activation.

A linear form of polyubiquitin chains was recently discovered in the NF-κB signalling pathway following TNF stimulation [53, 54]. In contrast to the linkage through internal lysine residues, linear ubiquitin chains exhibit a head-to-tail thread-like pattern in which the C-terminal glycine of ubiquitin is conjugated with the *N*-terminal methionine of another. The generation of linear ubiquitin polymers is catalysed by a distinct ubiquitin ligase machinery, named LUBAC (linear ubiquitin chain assembly complex), which is composed of two RING-between-RING domain-containing proteins, HOIL1 and HOIP [7, 55]. Emerging evidence shows that LUBAC is recruited to TNF receptor complexes upon TNF induction, and then conjugates linear ubiquitin chains to the regulatory subunit NEMO of the IKK complex [56, 57]. This activates the kinase activity of IKK and ubiquitin-dependent degradation of phosphorylated IκBα, thus enabling nuclear translocation of NF-κB dimers and downstream gene expression. Together, these studies establish the linear ubiquitin chain as an important post-translational modification in regulating TNF-induced NF-κB signalling.

### SHARPIN and NF-κB regulation

Dysregulated NF-κB activation is associated with a wide spectrum of inflammatory diseases [58–60]. The severe systemic inflammation combined with immune system defects in *Sharpin*^*cpdm*^ mice suggested that the mutation affects the regulation of NF-κB signalling. Recent reports indicate that this is the case [6, 8, 14, 16, 27, 28]. SHARPIN and HOIL1 share significant sequence similarity, including the UBL and NZF domains, leading to the hypothesis that SHARPIN may also be an adaptor component of the ubiquitin ligase complex LUBAC. Indeed, immunoprecipitation assays have shown that three putative complexes potentially exist: HOIP-HOIL1, HOIP-SHARPIN and HOIP-HOIL1-SHARPIN. Among these dimeric and trimeric complexes, HOIP is the indispensible catalytic component for linear polyubiquitination. In response to TNF, CD40L, IL1β and anti-LTβR, the canonical NF-κB activation, as indicated by IKK kinase activity, IκBα level and nuclear translocation of RELA, was found to be consistently impaired in SHARPIN-deficient splenic B cells, macrophages and mouse embryonic fibroblasts (MEFs). These results suggest a ubiquitous and selective role of SHARPIN in regulating classical NF-κB signalling downstream of distinct stimuli. Earlier studies identified NEMO as the substrate protein targeted by the HOIL1-HOIP complex in the TNF-induced NF-κB pathway [56, 57]. In line with this, LUBAC that contains SHARPIN as its adaptor component seems to function *via* similar mechanisms [6, 8, 14].

**Fig 1:**
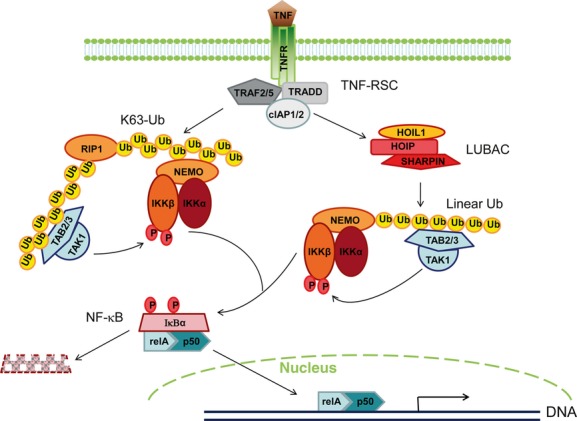
A proposed mechanistic model on how SHARPIN regulates TNF-induced NF-κB activation through linear ubiquitination-mediated mechanisms. TNF ligation induces TNFR1 trimerization and promotes the assembly of a multiprotein complex named TNF-RSC (TNF receptor signalling complex), which is composed of adaptor protein TRADD, TRAF2/5 and E3 ubiquitin ligases cIAP1/2. The signalling transduction then bifurcates into ‘TNFR-Lys63 Ub’ and ‘TNFR-linear Ub’ branches that are dependent upon different ubiquitin-dependent regulatory strategies. The first downstream ‘TNFR-Lys63 Ub’ cascade utilizes the E3 ligases cIAP1/2 to catalyse the formation of Lys63-linked ubiquitin chains to RIP1, which recruits and brings in spatial proximity the two kinase complexes TAK1-TAB2/3 and NEMO-IKKα-IKKβ. The activated kinase TAK1 then phosphorylates IKKα, which further phosphorylates IκBα to promote its proteasomal degradation. The other downstream ‘TNFR-linear Ub’ cascade employs linear ubiquitin chain-dependent mechanisms. TNF-RSC can function as a platform to attract LUBAC components, including HOIL1, HOIP and SHARPIN. LUBAC induces the formation of the linear ubiquitin chain to NEMO, which in turn recruits the kinase complex TAK1-TAB2/3. TAK1 then phosphorylates and activates the IKK complex similar to the ‘TNFR-Lys63 Ub’ pathway. Both ‘TNFR-Lys63 Ub’ and ‘TNFR-Linear Ub’ cascades converge at the IκBα-RELA-p50 complex that migrates into the nucleus for downstream gene activation after IκBα release and degradation.

On the basis of these results, a mechanistic model at molecular levels can be proposed for the physiological role of SHARPIN in regulating TNF-induced NF-κB regulation. Upon TNFR1 trimerization induced by TNF ligation, adaptor protein TRADD, TRAF2/5and E3 ubiquitin ligases cIAP1/2 are recruited to the cytoplasmic death domain of TNFR1, forming a multiunit complex named TNF-RSC (TNF receptor signalling complex). The ensuing signalling then diverges in two directions according to the primary type of the regulatory ubiquitin chains involved (Fig. 1). The first downstream pathway involves the E3 ligases cIAP1/2 catalysing the formation of Lys63-linked ubiquitin chain to RIP1, which concurrently recruits the kinase complexes TAK1-TAB 2/3 and NEMO-IKKα-IKKβ. Once in spatial proximity, the kinase TAK1 can phosphorylate IKKα, which in turn phosphorylates IκBα and targets it for proteasomal degradation. In contrast to the ‘TNFR-Lys63 Ub’ direction, linear ubiquitin chains are mainly utilized and conjugated to NEMO. All LUBAC components, HOIL1, HOIP and SHARPIN, bind ubiquitin chains of distinct types, albeit to varying degrees [6, 14, 56]. The Lys63-linked ubiquitin chain on cIAP1/2 appears to serve as the platform to recruit and bind LUBAC, which in turn adds the linear ubiquitin chain to NEMO. This additionally recruits the kinase complex TAK1-TAB 2/3 that can bind the linear form of ubiquitin chain [57], enabling its immediate kinase activity on the IKK complex similar to the ‘TNFR-Lys63 Ub’ pathway. Moreover, NEMO selectively recognizes linear ubiquitin chains through its UBAN domain [61, 62]. This indicates that the NEMO-IKKα-IKKβ complex binds linear ubiquitin chains on other NEMO molecules and subjects more IKKβ substrates to TAK1-mediated phosphorylation and activation, thereby amplifying the response. It is also possible that NEMO-IKKα-IKKβ complexes are induced to oligomerize, resulting in autophosphorylation and autoactivation of the IKKβ kinase. Both ‘TNFR-Lys63 Ub’ and ‘TNFR-Linear Ub’ pathways converge at the IκBα-p50-RELA complex that translocates into the nucleus upon IκBα release and degradation.

The working model implies that SHARPIN and NEMO are both novel components of the ‘TNFR-Linear Ub’ pathway. This is supported by the fact that *Sharpin*^*cpdm*^ mice and *Nemo*-deficient mice (*Ikbkg*^*tm1Mka*^ and *Ikbkg*^*tm1Mpa*^; both are mouse models for incontinentia pigmenti in humans) share many phenotypic similarities (Table 2). These mouse mutants have severe skin inflammation with increased epidermal hyperplasia and infiltration of granulocytes [63, 64]. At the cellular level, both *Sharpin*^*cpdm*^ and *Ikbkg*-deficient MEFs are highly sensitive to TNF-induced apoptosis [14, 63, 64]. Furthermore, the TLR2-induced transcriptome responses are similarly affected by *Sharpin* and hypomorphic *Ikbkg* mutations in macrophages and more importantly, SHARPIN and NEMO are direct interaction partners [28]. Despite these remarkable similarities, however, there are also notable phenotypic differences between the two mutants. For example, *Ikbkg*-deficient mice start to develop symptoms at 3–4 days after birth, whereas *Sharpin*^*cpdm*^ mice first show signs of disease at 3-4 weeks after birth. In addition, the *Ikbkg* mutation in macrophages appears to have a stronger effect on gene transcription than *Sharpin* mutations [28]. This could be explained by our proposed model showing that NEMO is participating in both ‘TNFR-Lys63 Ub’ and ‘TNFR-Linear Ub’ pathways, whereas SHARPIN is only involved in the ‘TNFR-Linear Ub’ pathway. In addition, the phenotype of *Sharpin* deficiency may be partially rescued by its functional counterpart HOIL1.

**Table 2:** Phenotypic comparisons between *Ikbkg* and *Sharpin* mutations in mice

	*Ikbkg* allelic mutations (*Ikbkg*^*tm1Mka*^ and *Ikbkg*^*tm1Mpa*^)	*Sharpin* mutation (*Sharpin*^*cpdm*^)
Genetic locus	Chr. X	Chr. 15
Embryonic lethality	Yes in males No in heterozygous females	No
Growth retardation	Yes	Yes
Onset of disease	3–4 days after birth	3–4 weeks after birth
Spleen	Decreased in size, no marginal zone	Enlarged in size, no marginal zone
Skin	Alopecia, inflammation with granulocytic infiltration, apoptotic keratinocytes	Alopecia, inflammation with granulocytic infiltration, apoptotic keratinocytes
Epidermis thickness	Increased	Increased
Dominant cytokines in skin	IL1α, IL1β, TNF, IFNG, TGFB1/2	IL4, IL5, IL13
Sensitivity to TNF-induced apoptosis	Increased	Increased
Melanin deposits	Yes	No

The proposed mechanistic model explains the NF-κB down-regulation in SHARPIN-deficient cells, as well as the phenotypic similarity between *Sharpin* and *Ikbkg* mutations [28, 65]. Nonetheless, it has raised several interesting questions that require additional investigations. First, why are both adaptor proteins HOIL1 and SHARPIN present in the LUBAC complex while maintaining similar E3 ligase functions? One possible reason could be that the presence of additional adaptor proteins synergistically enhances the tertiary stability of the tripartite complex. Indeed, absence of SHARPIN or HOIL1 resulted in destabilization of the other [6] and SHARPIN alone appears less efficient in complexing with HOIL1-HOIP [8]. It is also likely that the distinct adaptor proteins determine the specificity of downstream binding partner(s) and effector functions. Unlike *Sharpin*^*cpdm*^ mice, deletion of HOIL1 in mice does not cause overt pathology (Ref. [57] and K Iwai, personal communication), suggesting differential roles of SHARPIN and HOIL1 *in vivo*. Their binding affinity for different types of ubiquitin chains also vary slightly, potentially due to the fact that HOIL1 contains two additional RING domains and one IBR domain that are not present in SHARPIN [6]. This may result in differential interaction preferences for downstream ubiquitin-conjugated proteins. It should be noted that HOIL1 itself also functions as an E3 ligase, as demonstrated in the metabolism of oxidized IREB2 in iron-rich cells [66]. Another intriguing question is why TNFR1 activation uses two parallel pathways that utilize linear and Lys63-linked ubiquitin chains, considering that both pathways converge on IκBα polyubiquitination and degradation. A possible explanation is that this apparent duplication provides a multilayered regulatory mechanism that fine-tunes the ultimate strength of NF-κB activation. Furthermore, linear and Lys63-ubiquitinated cascades may be differentially involved in other signalling pathways. For example, SHARPIN-mediated ubiquitination may also regulate TLR2-induced responses [28]. One additional outstanding question is how the working model fits the inflammatory phenotype in *Sharpin* mice. NF-κB signalling activates many anti-apoptotic genes, [67–69]. Interestingly, the epidermis of *Sharpin* mice contains apoptotic keratinocytes and SHARPIN-deficient MEFs *in vitro* and keratinocytes *in vivo* were extremely susceptible to TNF-induced apoptosis. Deletion of TNF in *Sharpin* mice partially corrected the phenotype and completely rescued the cutaneous inflammation [14]. It was suggested that physiological concentrations of TNF induce apoptosis in keratinocytes resulting in the release of pro-inflammatory signals that initiate and amply an inflammatory response in *Sharpin*^*cpdm*^ mice. However, light microscopic observations suggest that the inflammation in the dermis precedes the epidermal changes [12] and the exact pathogenesis of the dermatitis as well as the inflammation in other organs remains to be determined.

### SHARPIN and β1-integrin signalling

A recent study identified SHARPIN as an important regulator of β1-integrins [2]. Integrins are type I heterodimeric transmembrane proteins involved in adhesion to other cells and to extracellular matrices [70, 71]. They have important functions in biological processes such as cell migration, proliferation and survival and in immune and inflammatory responses [72]. Activation of integrins can be induced by ligand binding (‘outside-in’) resulting in recruitment of adaptor proteins to the short cytoplasmic domains and clustering of the integrins. In addition, intracellular signals can alter the conformation of the integrins causing an increase in affinity (“inside-out” signalling). SHARPIN binds to conserved sequences in the α1-, α2- and α5- subunit of β1-integrins inhibiting activation of the integrins [2]. Increased β1-integrin activity was observed in keratinocytes and leukocytes of *Sharpin*^*cpdm*^ mice and suppression of SHARPIN in human peripheral blood leukocytes increased the migration on fibronectin-coated surfaces. This role of SHARPIN appears to be ubiquitin- and LUBAC-independent. The correlation between these results and the inflammatory phenotype of *Sharpin*^*cpdm*^ mice is not clear.

### SHARPIN in lymphoid organogenesis

Aside from the systemic inflammation, another intriguing feature of the *cpdm* phenotype is the defective development of secondary lymphoid tissues. The underlying pathological mechanisms are still poorly understood. Lymphoid organogenesis is thought to preferentially involve the alternative NF-κB signalling mediated by the RELB/p52 heterodimer. This notion is supported by many lines of evidence in transgenic mice with impaired activation of alternative NF-κB signalling, mainly achieved by genetic inactivation of *Nfkb2*, *RelB* or *Nik* [33]. Strikingly, these distinct lines of targeted mutant mice exhibit similar defects in the architecture of the spleen (lack of germinal centres, follicular dendritic cells and marginal zones) and absence of LNs and Peyer's patches [33]. However, processing of p100 to generate p52 remains unaffected in *Sharpin*-deficient B cells and MEFs when stimulated with CD40L, BAFF, TWEAK and anti-LTBR antibody [8, 14], implying that alternative NF-κB signalling may not play a role in the *Sharpin*^*cpdm*^ phenotype. There are phenotypic differences between *Sharpin*^*cpdm*^ mice and *Nfkb2*^*tm2Brv*^ (*Nfkb2*^−/−^), *Relb*^*tm1Brv*^ (*RelB*^−/−^) or *Nik*^*aly*^ (*Nik*^−/−^) mice. *Sharpin*^*cpdm*^ mice have both peripheral and mesenteric LNs [13] in contrast to *Nfkb2*^−/−^, *RelB*^−/−^ or *Nik*^−/−^ mice. Given the attenuated TNF-induced NF-κB activation SHARPIN-deficient mice, we hypothesize that the classical and alternative NF-κB signalling may have differential roles in the *de novo* development of secondary lymphoid tissues. The RELB/NFKB2 heterodimer of NF-κB is preferentially involved in initiating lymphoid organogenesis. In contrast, the RELA/NFKB1 heterodimer of NFKB seems more important for maintaining the proper architecture of lymphoid tissues. This is supported by the normal lymphoid organogenesis in *Nfkb1*^−/−^ mice albeit with reduced numbers and size of Peyer's patches [33]. Mice with *Ikbkg* mutations develop a thymus and spleen, although highly disorganized and considerably smaller than wild-type controls [63].

## Concluding remarks

The complex phenotype of *Sharpin*^*cpdm*^ mice indicates that SHARPIN plays an important role in inflammation and the development of the immune system. Molecular studies have clearly demonstrated that SHARPIN is an essential regulator that modulates canonical NF-κB cascade through linear ubiquitin-mediated mechanisms. However, how the absence of SHARPIN leads to chronic type 2 inflammation in multiple organs and defects in the development and micro-architecture of lymphoid organs remains to be determined. SHARPIN also acts as an inhibitor of β1-integrins activation [2] and down-regulates the activity of tumour suppressor protein PTEN [73, 74]. Clearly, the biological function of SHARPIN is only beginning to be unravelled.
